# A Review of the Effect of Preparations from Vegetables of the *Asteraceae* Family and *Cucurbitaceae* Family on the Cardiovascular System and Its Diseases

**DOI:** 10.3390/nu14173601

**Published:** 2022-08-31

**Authors:** Agata Rolnik, Beata Olas

**Affiliations:** University of Lodz, Department of General Biochemistry, Biology and Environmental Protection, 90-236 Lodz, Poland

**Keywords:** antiplatelet activity, *Asteraceae*, cardiovascular disease, cardiovascular system, *Cucurbitaceae*, oxidative stress

## Abstract

Cardiovascular (CV) system dysfunction can result in the development of cardiovascular diseases (CVDs), a key cause of death around the world. For many people, the most common treatment choices are still based on various plants used in traditional and folk medicine. Interestingly, many of these plants demonstrate various biological activities and pro-health properties; as such, there has been growing scientific interest in their use as medicines, including treatments for CVDs. Due to their varied biological properties, including anti-inflammatory and anticancer potential, the members of the *Asteraceae* and *Cucurbitaceae* have long been used in traditional medicine. These properties are believed to derive from the chemical composition of the plants, which includes various flavonoids, phenolic acids, and terpenes. Although many of their pro-health properties have been well described, their effect on the cardiovascular system and CVDs remains unclear. The present work reviews the current literature about the effects of preparations of vegetables of the *Asteraceae* and *Cucurbitaceae* families on the cardiovascular system and CVDs. Various species from the two families demonstrate antioxidant and antiplatelet activities in vitro and in vivo, which play key roles in the prophylaxis and treatment of CVDs. Additionally, some species have been evaluated for their anticoagulant activity. This review also describes the biological properties of these vegetables and discusses their anti-hyperlipidemic action, and their potential for obesity prevention and body weight control.

## 1. Introduction

The cardiovascular system comprises a cardiac pump, a circulatory network formed from the endothelium, and blood together with its active components. The main organ is the heart, which is composed of four morphologically and functionally distinct chambers. Its main function is to propel deoxygenated blood to the lungs and then distribute the oxygenated blood across the body [1]. The cardiovascular system also plays a key role in hemostasis: a set of processes responsible for closing damaged blood vessels, maintaining the blood in a fluid state, and removing blood clots after restoration of vascular integrity.

The critical element of hemostasis is the blood platelet, which closes the injured vessel in the first wave of hemostasis and then facilitates the coagulation process. However, pathological platelet activation can result in the formation of a clot in a healthy vessel, leading eventually to the development of various cardiovascular diseases (CVDs) [2]. Platelets are one of the main factors in the development of heart failure and stroke. Their basic function is to prevent excessive bleeding by rapidly binding to damaged blood cells and aggregating to form thrombi. Unfortunately, their unnecessary activation can lead to aggregation, for example at the site of atherosclerotic plaque rupture, and consequent thrombus formation. These blockages promote the development of atherothrombotic disease. As such, many cardiovascular therapies are based around anti-platelet medication [3].

The coagulation process itself employs a group of zymogens, which are converted to active enzymes when necessary, resulting in the formation of thrombin, followed by the transformation of fibrinogen to fibrin. In a pathological state, this process can occur in a healthy vessel, leading to the creation of an unwanted clot. Such problems involving blood thickening are usually treated with anticoagulants, which slow down blood clotting mechanisms [4]. Most anticoagulants are available as drugs, but some occur in plants as secondary metabolites; among these, the strongest anticoagulant activity has been demonstrated for flavonoids, coumarins, and saponins. The presence of terpenes, tannins, saponins, and flavonoids in plant extracts seems to have a significant influence on reducing the time of plasma coagulation and blood clotting [4].

CVDs are a major cause of death around the world. The World Health Organization estimates the total mortality for CVD-related causes in 2019 to be around 18 million. In Europe alone, they account for around 46% of total deaths. In the USA, according to the Centers of Disease Control and Prevention, a heart attack occurred every 40 s in 2020. These diseases can easily develop throughout the population, especially among adults, and are often untreated in their early stages. The most common cardiovascular diseases are heart failure, stroke, coronary heart disease, myocardial infraction, and hypertension. Of these, stroke and coronary heart disease are the most common causes of death in the West. Both develop in response to a lack of oxygen in the brain and heart. Stroke can often be caused by blood coagulation, leading to the formation of a clot that prevents blood flow to brain tissue [1].

Other key risk factors in the development of CVDs are diabetes mellitus, high cholesterol level, cigarette smoking, and a diet rich in saturated fats. In addition, dysfunctions in the lipid metabolism are also strongly connected with atherosclerosis initiation and progression, due to their potential to induce oxidative stress and inflammation. Oxidative lipids take part in each stage of atherosclerosis, including fatty streak formation, lesion establishment, and plaque rupture [5].

In addition, low-density lipoprotein cholesterol (LDL), one of five major types of lipoproteins, also plays a crucial role in CVD development. LDL is able to penetrate the endothelium of arterial walls and become oxidized. This promotes inflammation and causes injury to the overlying endothelium and surrounding smooth muscle cells. A persistent high level of LDL is correlated with the progression of lipid-rich plaques [6]. Hypertension, like obesity is also one of the most often occurring in diverse parts of world [7].

Although modern medicine can be effective, it is not always accessible for people living in low- and middle-income countries. In such cases, prophylaxis is particularly important. Indeed, poor diet is believed to be one of the greatest risk factors in the development of CVDs. Fortunately, many everyday plant-based dietary components contain physiologically active components that can positively influence the function of the cardiovascular system and CVDs; studies have shown that they have various cardioprotective effects, including antioxidant, anti-platelet, and anticoagulant properties [8].

Plants have played a key role in the development of medicine, from the traditional treatments, when plants were the only available therapy, to more modern ones which draw on their secondary metabolites and biological activity, to create more effective drugs. Even today, the use of medical plants is often considered the same as primary health care. The World Health Organization (WHO) estimates that almost 80% of population relies on traditional, plant-based medicine in one form or another [4], and its use is growing globally, due to its effectiveness in certain diseases [7].

Both the *Cucurbitaceae* and *Asteraceae* are large plant families whose vegetables are popular in diets around the world. They are significant components of both folk and modern medicine, and are known to play a positive role in human health [9,10]. However, little is known of their effect on the cardiovascular system and CVDs. Some review papers clearly indicate that certain vegetables, including garlic (*Allium sativum* L.), onion (*Allium cepa* L.), and beetroot (*Beta vulgaris* L.) have beneficial effects on the cardiovascular system and CVDs [11,12]. Therefore, this present review examines the available literature regarding the biological potential of vegetables from the *Cucurbitaceae* and *Asteraceae* families, with particular regard to their antioxidant, anti-platelet, anticoagulant potential, as well as their anti-hyperlipidemic action and possible value in obesity prevention and body weight control, based on various in vitro and in vivo trials. In addition, both families have been chosen for this review due to their diverse used in diets around the world. Their representatives, like cucumber or pumpkin are one of the most often eaten vegetables in every part of the world. They are also easy and cheap to grow. The review is based on studies identified in various electronic databases. The last search was run on 30 May 2022. The main search criteria covered a combination of the following terms: “vegetable”, “*Cucurbitaceae*”, “*Asteraceae*”, “cardiovascular system” and “cardiovascular diseases”.

## 2. Oxidative Stress and Cardiovascular System; the Role of Antioxidants

Oxidative stress is one of the main risk factors in cardiovascular disease. At the cellular level, it occurs due to the release of free radicals by endothelial and vascular smooth muscle cells and is one of the prime pathogenic factors in the development of cardiovascular diseases. Biological systems generate reactive oxygen species (ROS), such as superoxide radicals, hydrogen peroxide, and hydroxyl radicals, as metabolic by-products [13]. At low levels, they have key roles in redox signaling, which is part of the cellular signaling system. The redox system is defined as specific and reversible oxidation/reduction modification of components in cellular signaling, such as cell growth or gene expression [14]. However, a pathological state, defined as prominent level of ROS, leads to oxidative stress in cells. Free radicals cause oxidative damage in various cellular macromolecules, including DNA, proteins, and lipids. They are also believed to play a vital role in the progressive pathology of atherosclerosis associated with endothelial dysfunction, and are known to be associated with pro-inflammatory and prothrombotic status. Oxidative stress is related with various CVDs risk factors, including hypertension, hyperlipidemia, obesity, diabetes mellitus, and cigarette smoking [13,14].

Two sets of antioxidant defenses protect biological systems from the toxic effect of ROS, one based on non-enzymatic antioxidants, and the other on enzymes such as catalase, glutathione peroxidase, and superoxide dismutase. Many plants are sources of such antioxidants [15], whose activity mostly comes from phenolic compounds. However, the bioavailability of these phenolic compounds in humans depends mostly on the activity of the gut microflora, where they undergo methylation, sulfation, and glucuronidation [13]. These polyphenols mainly act by scavenging free radicals, but they can also prevent free radical formation, chelate metal ions, and improve the endogenous antioxidant system. They can also prevent endothelial dysfunction and inflammation caused by oxidative stress. For example, the polyphenols in red wine can reduce cholesterol level and improve cardiac performance [14]. Another group of secondary metabolites which have demonstrated strong antioxidant activity are the flavonoids. Their activity is mostly dependent on their molecular configuration, the position, and the number of hydroxyl groups; they are believed to exert their antioxidant by three different mechanisms: eliminating ROS, interacting with enzymes that control ROS production, and increasing the degree of protection of the antioxidant system. All three mechanisms influence the cardiovascular system by interfering with lipid oxidation and decreasing blood platelet activation and aggregation [16].

Vitamins are also considered to be strong antioxidants. Ascorbic acid (vitamin C) participates in vascular and connective tissue maintenance, collagen biosynthesis, and iron absorption. Its antioxidant activity is based on its free radical scavenging ability and ability to protect membrane phospholipids from oxidation. Another vitamin, vitamin E, belonging to the tocopherols, also exerts antioxidant activity; it is believed to act by protecting cell membranes against free radical damage promoted by low-density lipoprotein, and positively influencing biomarkers of oxidative stress. In addition, carotenoids, the precursors of many vitamins, including vitamin A, exert antioxidant activity by absorbing excess ROS through electron-rich conjugated double-bond systems [15,17].

## 3. Pharmacological Characteristics of the *Asteraceae* Family of Plants

The *Asteraceae* family, also known as the Compositae, contains over 25,000 species worldwide, including chamomile (*Chamaemelum nobile*), chicory (*Cichorium intybus*), lettuce (*Lactuca sativa*), dahlias (*Dahlia pinnata*), and dandelion (*Taraxacum officinale*). Many of these species are widely distributed worldwide, but the largest concentration occurs in temperate, cold-temperate, and subtropical regions [7,10]. Most plants from this family tend to have hairy and aromatic leaves and flat clusters of small, colorful flowers on the top of the stem, which results in their application as garden plants. In addition to their decorative use, most species also have therapeutic applications. Both dandelion and chamomile have strong anti-inflammatory and diuretic activities. Chicory was a traditional medicine for liver disorders due to anti-inflammatory activity [18,19]. In addition, the aqueous extract of *Achillea kellalensis* has shown significant wound-healing activity in rats; treatment results in faster healing in rats compared to controls, which is attributed to the activity of flavonoids [19]. Similarly, *Chromolaena odorata* has also demonstrated considerable wound-healing ability: in traditional medicine, the leaves were used to create a paste, which was applied topically on the wound [19].

*Lactuca sativa*, also known as lettuce, is considered a particularly important leafy vegetable, due to its role in everyday diets. However, it is also a source of various bioactive metabolites, including anthocyanins and sesquiterpene lactones, which are believed to demonstrate various pharmacological actives, including antioxidant and antibacterial properties [20]. Moreover, *Cynara cardunculus* (artichoke) is often used as dietary prebiotic, but it has also demonstrated hepatoprotective, hypolipidemic, and hypoglycemic properties [21].

In addition, *Achillea tenuifolia* extract suppresses lipid peroxidation during the initial stages of peroxidation. Its activity has been determined using a linoleic acid system, with peroxidation measured using thiocyanate. Moreover, methanolic extract from *Artemisia absinthium* at a concentration of 25 to 100 μg/mL demonstrated strong scavenging activity on superoxide anion radicals produced by PMS-NADH (nicotinamide adenine dinucleotide phosphate) system. This extract also decreased the level of thiobarbituric acid reactive substances (TBARS) when administered orally at 100 mg/kg of body weight [7]. *T. officinale* also demonstrated strong antioxidant activity that correlated with its chemical composition. The roots, leaves, and petals are rich sources of phenolic compounds and terpenes; in vitro studies on human plasma found both dandelion leaf and petal extracts inhibited lipid peroxidation in plasma induced by H_2_O_2_/Fe^2+^ (the donor of hydroxyl radicals). The two extracts also inhibited protein carbonylation and oxidation of protein thiols in plasma; however, stronger antioxidant activity has been noted for the petals, which are rich in phenolic acids, than the leaves, which are sources of flavonoids [22].

Lis et al. [23] analyzed the antiplatelet activity of five dandelion root fractions (A-E) at concentrations of 10–50 μg/mL in vitro. Their antiplatelet activity appears to be mostly based on inhibiting blood platelet activation, i.e., by preventing adhesion to fibrinogen or collagen. For thrombin-activated platelets, fractions B, C, D, and E inhibited adhesion to collagen, but only fraction C, D, and E prevented adhesion to fibrinogen. For ADP-activated platelets, fractions A, C, D, and E prevented adhesion. Each fraction has a unique chemical composition; for example, fraction C is rich in hydroxyphenylacetate inositol esters [23].

An in vivo study examined the effects on three groups of male albino Wistar rats: one group received dandelion leaf extracts, another dandelion petal extracts, and the other a normal diet [24]. The findings indicate a decrease in oxidative stress biomarkers in blood plasma, including protein carbonylation, in the first and second group compared to controls. Other studies found rats supplemented with leaf or petal extract had lower levels of lipid in blood plasma [24].

Interestingly the members of the *Asteraceae* contain various compounds that demonstrate anticoagulant activity. *T. officinale* shows anticoagulant activity, due to its high phenolic compound content. For example, five different fractions (A-E) obtained from *T. officinale* roots prolonged thrombin time, a parameter of coagulation, in human blood plasma in vitro. However, the activity varied depending on the fraction type and chemical composition. The strongest effect was demonstrated by the chlorogenic acid-enriched fractions and chicoric acid-enriched fractions [23,25].

Some members of the *Asteraceae* show anti-hyperlipidemic activity. A daily dose of 400 mg/kg body weight *Achillea arabica* extract was found to reduce hyperlipidemia in rats fed a high-fat diet [7]. Another example of an *Asteraceae* plant with antihyperlipidemic activity is *Ageratum conyzoide*. The leaf and root extracts reduce the level of lipids in the serum in rats supplemented with 100 mg/kg of body weight of extract in vivo. The rats also demonstrated lower total cholesterol levels and elevated amounts of high-density lipoprotein (HDL) cholesterol [26].

### 3.1. Vegetables from the Asteraceae Family and Their Effect on Oxidative Stress

Various plants from the *Asteraceae* family have demonstrated strong antioxidant activity in vitro and in vivo [10]. This activity often depends on their phytochemical concentrations, such as their flavonoid, polyphenol, and tannin levels [27]. Indeed, it has been proposed that the presence of high levels of phytochemicals from each group is responsible for the free radical scavenging activity of *Cichorium intybus* [28]. In addition, the presence of secondary metabolites such as sesquiterpene lactones and caffeoyl derivatives, is believed to be responsible for the antioxidant activity demonstrated in artichoke and burdock [29].

#### 3.1.1. In Vitro Studies

Rolnik et al. [30] attribute the antioxidant activities of selected vegetables from the *Asteraceae* family to their high polyphenol and phenolic acid content. A qualitative analysis of four vegetables from the *Asteraceae* family (chicory, green lettuce, red lettuce, and Jerusalem artichoke) revealed the presence of three main classes of metabolites, viz., phenolic acids, flavonoids, and sesquiterpene lactones, all of which show demonstrated potential antioxidant activity. The antioxidant activity of each vegetable was confirmed by TLC-DPPH• (thin layer-chromatography with 2.2-diphenyl-1-picrylhydrazyl), and all vegetable preparations showed activity against chlorogenic acid. Moreover, when applied at concentrations of 1–50 µg/mL, all vegetable preparations demonstrated antioxidant activity in human plasma, reducing the level of plasma protein carbonylation and plasma protein thiol oxidation in plasma treated with H_2_O_2_/Fe^2+^, a hydroxyl radical donor. Additionally, the chicory, green lettuce, and sunchoke vegetable preparations altered the level of lipid peroxidation in plasma induced by H_2_O_2_/Fe^2+^. The highest antioxidant capability was demonstrated by red lettuce, which possessed the highest phenolic acid content, but the highest antioxidant activity in human plasma was noted for chicory and Jerusalem artichoke [30] (Table 1).

The antioxidant activity of *Cichorium intybus* was also confirmed by Epure et al. [27] in vitro. The aerial parts were air-dried and used for methanol extraction with two variants: 5 g of powder plant with 50 mL of 70% methanol at 60 °C, and 50 g in 500 mL of 70% methanol at room temperature for seven days. The antioxidant properties of the methanol extracts were measured by free radical scavenging 2,2-diphenyl-1-picrylhydrazin (DPPH) and FRAP (ferric-reducing antioxidant power) tests in serum. Although both extracts yielded good FRAP scores, the first variant appeared to be more effective. This free radical scavenging ability may be due to the high polyphenol content [27] (Table 1).

Abdalla et al. [20] examined the antioxidant activity of three different lettuce cultivars (green butterhead, multi-leaf lettuce, and red multi-leaf lettuce) using free radical DPPH assay. The extracts were prepared at 20 mg/mL concentrations. Although all methanol extracts showed promising radical scavenging activity, red lettuce appeared to be the strongest, due to its high anthocyanin level, especially cyanidin 3-O-galactoside [20] (Table 1).

Koukoui et al. [31] evaluated the in vitro antioxidant activity of *Launaea taraxacifolia*, leafy vegetables. Leaf extracts (1–20 μg/μL) were added to PLB985 cells stimulated with phorbol myristate acetate to increase ROS production. The extract was found to decrease ROS production, suggesting that *L. taraxacifolia* has antioxidant activity [31] (Table 1).

#### 3.1.2. In Vivo Studies

However, only in vivo study has examined the antioxidant activity in *Asteraceae*. The activity of *Cichorium intybus* extract was estimated based on malondialdehyde (the marker of lipid peroxidation) and total thiols content in 25 adult male Winstar albino rats. The rats were divided into five groups: one negative control, one isoprenaline group, and three groups receiving *C. intybus* tincture (CHT), viz. CHT 100%, CHT: solvent 1:1 = 50%, CHT: solvent 1:3 = 25%). Oxidative stress biomarkers were found to be reduced in the CHT groups, indicating antioxidant activity [27] (Table 1).

### 3.2. Vegetables from the Asteraceae Family and Their Antiplatelet Activity

Many species of *Asteraceae* show strong antiplatelet activity, and this appears to be correlated with their chemical composition. The *Asteraceae* are especially rich in polyphenols, which can influence cardiovascular properties by inhibiting platelet activation, adhesion, and aggregation, and can even target the specific thrombogenic pathway [32,33].

#### 3.2.1. In Vitro Studies

One study examined the antiplatelet activity of four preparations (1–50 µg/mL) from *Asteraceae* vegetables (chicory, green lettuce, red lettuce, and Jerusalem artichoke) in vitro. The study used various markers of blood platelet activation, including platelet adhesion to collagen and fibrinogen, and arachidonic acid metabolism. All blood platelets were isolated from fresh human blood and were activated by thrombin or ADP. All preparations significantly inhibited adhesion to fibrinogen for all platelets. They also significantly inhibited collagen adhesion in resting and thrombin-activated platelets when used at the highest concentration (50 µg/mL); in addition, the Jerusalem artichoke and red lettuce preparations were found to be more potent than the chicory and green lettuce preparations [33]. All the tested preparations also inhibited arachidonic acid metabolism in the thrombin-activated blood platelets. Their antiplatelet activity appears to depend on the phenolic profile, including the presence of 1,3-dicaffeoylquinic acid, 5-caffeoylquinic acid, flavonoids such as quercetin derivatives, and sesquiterpene lactones such as lactucin derivatives. All these compounds may have the ability to modulate platelet activation. Interestingly, none of the preparations caused any significant changes in the resting or ADP/collagen-activated platelets with regard to their platelet activation markers, such as P-selectin or CD62P [33].

#### 3.2.2. In Vivo Studies

Schumacher et al. [34] analyzed the antiplatelet activity of coffee made from chicory, a preparation with high levels of caffeic acid, a polyphenol known to inhibit platelet aggregation. Briefly, 27 healthy volunteers drink 300 mL of 20 g ground chicory coffee each morning. The antiplatelet activity was measured with platelet aggregation in platelet-rich plasma taken from the participants. It was found that the chicory coffee inhibited collagen-induced platelet aggregation after eight days of regular consumption [34] (Table 1).

### 3.3. Anticoagulant Activity of Asteraceae Vegetables

Rolnik et al. [30] evaluated the anticoagulant activity of preparations (1–50 µg/mL) from four vegetables from the *Asteraceae* family, including chicory, green lettuce, red lettuce, and Jerusalem artichoke in vitro. The anticoagulant properties were evaluated based on coagulation time (including prothrombin time, thrombin time and activated partial thromboplastin time) in human plasma after 30 min incubation at 37 °C with the preparation. The longest prolongation of thrombin time was observed for Jerusalem artichoke preparation at the highest concentration; however, the results were not statistically significant [30]. None of the four vegetables demonstrated any significant differences in their effect on thrombus formation under flow conditions in human blood compared to controls, indicated by a lack of change in the area under the curve (AUC). The results showed little or no anticoagulant activity (Table 1) [33]. No in vivo studies have yet been performed of the effect of these vegetables on the coagulation process.

## 4. Characteristics of the Biological Properties of Plants from the *Cucurbitaceae* Family

The *Cucurbitaceae* family is one of the most diverse plants families worldwide, with 800 species flourishing in different environmental conditions. Over 300 species are used by humans in ways ranging from food and medicine to decorations. The cucurbits comprise a mixed group of fruits and vegetables, with the most common being pumpkin, cucumber, and watermelon [35,36]. Many family members are found in everyday diets around the word; for example, *Cucurbita pepo* can be added to soups, curries, and even pies, while cucumber is mostly eaten fresh as a main ingredient of salads [37].

Cucurbit seeds are considered good sources of protein and vitamins. However, despite being mostly considered as foods, cucurbits also have important physiological benefits on human health, including antioxidant, anti-inflammatory and hepatoprotective activities [27].

Many species are used in traditional medicine. Pumpkin seeds have been recommended as therapy for tapeworm or kidney disorders, and bitter apple has been used as purgative and analgesic agent in Arabian countries. These properties are of course dependent on the chemical compounds present in the plant [38]. Cucumber is believed to support wound healing and ease skin infections and inflammations due to its tannin and phytosterol content. In addition, cucumber is considered a cooling and hydrating food in summer, due to its high water content. Pumpkin has been recommended to increase the appetite, and the seeds have often been used as remedies for fever, bronchitis, and sore chest [39]. *Momordica charantia*, also known as bitter melon, has long been used in traditional medicine, due to its varied pharmacological and nutritional properties, and is regarded as a treatment for diabetes and inflammatory diseases. It has been found that *M. charantia* extract can help reduce glycaemia in type 2 diabetic patients [40].

Various compounds present in *Cucurbitaceae* family members are known to influence the cardiovascular system; two examples are saponins and cardiac glycoside, which are both used in treating heart disease [9]. For example, saponins can stop bleeding by increasing blood coagulation. Cucurbitacins, present only in some cucurbit species, can inhibit cardiac hypertrophy by increasing autophagy among cardiomyocytes [41].

Iman et al. [42] found *Melothiria maderespatana* extracts (100 μg/mL, 200 μg/mL, 400 μg/mL, and 500 μg/mL) to have antiplatelet activity against ADP-induced platelet aggregation in vitro. The strongest effect was obtained by the 400 μg/mL extract. The antiplatelet activity was found to be correlated with the coumarin and flavonoid content of the fractions [42].

Butanol extract of *Bryonia dioca* roots also demonstrated strong free radical scavenging potential which positively correlated with its flavonoid and polyphenol content. Although the plant itself is toxic, the flowers and leaves can cause inflammation, and the skin and juice cause intense gastrointestinal irritation, the seeds are considered safe to eat [43].

### 4.1. Vegetables from the Cucurbitaceae Family and Their Effect on Oxidative Stress

#### 4.1.1. In Vitro Studies

Rolnik et al. [44] found extracts (1–50 µg/mL) from five selected cucurbit vegetables (pumpkin, zucchini, cucumber, white and yellow pattypan squash) to demonstrate antioxidant activity in human plasma treated with H_2_O_2_/Fe^2+^ in vitro. All preparations inhibited plasma lipid peroxidation and demonstrated antiradical properties based on the DDPH test. However, the yellow pattypan squash showed the strongest antioxidant activity; this was the only preparation that inhibited thiol group oxidation. The strong antioxidant activity of pattypan squash was correlated with high levels of benzoic acid derivatives and phenylpropanoid glycoside in its composition [44]. It is known that phenylpropanoid glycosides inhibit the oxidation of lipoprotein by metal ion chelation and free radical scavenging due to the presence of phenylpropanoid and phenylethanoid groups. The antioxidant activity of the benzenoid acid derivate has been attributed to the presence of a blocked hydroxyl group in its structure [45].

The antioxidant activity of pumpkin is also connected with its phenolic acid composition, as demonstrated by DPPH free radical assay. Commercially available pumpkin seeds were extracted in four different solvent fractions: water, acetone, methanol, and ethyl-acetate. The antioxidant activity of the fractions was dependent on phenol concentration, with the strongest scavenging activity demonstrated by the fractions with the highest phenolic contents: water and methanol [46]. In addition, 10 mg/mL aqueous and butanol extracts of *Citrullus colocynthis* fruits were found to have antioxidant activity based on FRAP and DPPH assay [43].

Yasir et al. [47] analyzed the antioxidant properties of selected *Cucurbitaceae* seeds by spectrophotometric DPPH assay. The study evaluated extracts from fruits of *Momordica dioica*, *Cucumis melo var. agrestic,* and *Citrullus colocynthus* L. in the concentration range 1–5000 µg/mL. All demonstrated some free radical scavenging activity, which was strongly correlated with the level of polyphenols in their chemical composition [47].

The antioxidant activity of *Momordica charantia* methanol and chloroform extracts in serum were analyzed in vitro by TBARS and free radical scavenging assay. Both extracts were prepared at the same concentration: 500 g of dry whole fruits were dissolved in 5 L of organic solvent. The methanol extract was found to have the strongest free radical scavenging activity, and greatest ROS inhibition, based on the TBARS assay. Both extracts demonstrated significantly higher inhibition than the controls, suggesting that both have antioxidant activity [48] (Table 1).

#### 4.1.2. In Vivo Studies

Dallak [49] analyzed the antioxidant activity of *Cutrullus colocynthis* extract in 24 diabetic albino rats with alloxan-induced hyperlipidemia in vivo. The extract was orally administrated at a dose 300 mg/kg of body weight. Liver tissues were taken and tested for lipid peroxidation biomarkers, such as TBARS and lipid hydroperoxides. The results suggest that the extract has antioxidant activity [49].

Xia et al. [50] examined the antioxidant activity of *C. ficifolia* fruits in streptozotocin-induced diabetic rats based on lipid peroxidation level in blood. Briefly, 24 male Sprague-Dawley rats were divided into four groups: normal controls, untreated diabetics, diabetic rats treated with *C. ficifolia* fruit extract, and normal rats treated with *C. ficifolia* fruit extract. Both groups treated with *C. ficifolia* fruit extracts were force-fed the dissolved extract (300 mg/kg body weight) in 2 mL of water each day. Lipid peroxidation level was measured by TBARS method. A lower level of oxidative stress was noted in the serum of the group supplemented with *C. ficifolia* fruit extract, indicating antioxidant activity [50] (Table 1).

### 4.2. Vegetables from the Cucurbitaceae Family and Their Antiplatelet Activity

#### 4.2.1. In Vitro Studies

Phenolic compounds are strongly implicated in the antiplatelet activities observed for preparations from the *Cucurbitaceae* family [38]. Rolnik et al. [51] evaluated the antiplatelet activity of extracts (1–50 µg/mL) from selected cucurbit vegetables (pumpkin, zucchini, cucumber, white and yellow pattypan squash) on blood platelets isolated from fresh human blood. The extracts were found to inhibit platelet adhesion to collagen or fibrinogen. Among thrombin-activated blood platelets, three preparations, viz., pumpkin, cucumber and yellow pattypan squash, inhibited collagen adhesion, while four (pumpkin, zucchini, white and yellow pattypan squash) prevented fibrinogen adhesion. Additionally, all tested extracts inhibited arachidonic acid metabolism in all platelets, and GPIIb/IIIa exposure on 10 µM ADP-activated platelets, which also is related to platelet activation [51] (Table 1).

#### 4.2.2. In Vivo Studies

Rajput et al. [52] found *Lagenaria siceriaria* to prevent ADP-induced platelet aggregation in Swiss albino mice in vivo. *L. siceriaria* extract was administrated orally at doses of 250, 5000, and 1000 mg/kg of body weight. The mice were divided in three groups: the first received the extracts, the second (control) received saline, and the third (control) received aspirin at 20 mg/kg of body weight. Extracts from *L. siceriaria* significantly inhibited platelet aggregation, suggesting antiplatelet activity; the degree of inhibition was correlated with the concentration of flavonoids, including kaempferol [52] (Table 1).

### 4.3. Anticoagulant Activity of Cucurbitaceae Vegetables

The antiplatelet activity of vegetables from the *Cucurbitaceae* family is based on their anticoagulant effect. This is commonly evaluated using a total thrombus-formation analysis system in freshly collected human whole blood (T-TAS technique). In one study, the strongest antiplatelet activity yellow was observed for pattypan squash extract (50 µg/mL), and this was attributed to its high concentrations of phenylpropanoid glycoside, a compound known for its antiplatelet activity [51].

*M. charantia* has been demonstrated to have anticoagulant activity in vitro. A study examined the anticoagulant activity of 0.5–5.0 μg/mL crude extract in platelet-poor plasma isolated from goats. Heparin and warfarin were used as positive controls. The study measured prothrombin time and activated partial thromboplastin time, and determined inhibitory thrombin and factor Xa-stimualted platelet aggregation. The tested extract was found to decrease platelet aggregation and anticoagulant activity [53] (Table 1).

## 5. Anti-Hyperlipidemia Effects of Preparations from Vegetables of the *Asteraceae* Family and *Cucurbitaceae*

Atherosclerosis can be caused by hyperlipidemia, a condition where lipids form plaques that harden the arteries, thus narrowing them and blocking blood flow. Antihyperlipidemic treatments are typically aimed at reducing the triglycerides and low-density lipoprotein (LDL) levels, and total cholesterol content [7]. *Cucurbitaceae* family members have been found to demonstrate antihyperlipidemic activity [31].

For example, *Citrullus colocynthis* was observed to lower serum triglyceride and cholesterol levels. Extracts from aerial parts also decrease glucose levels and enhanced glycogen formation in streptozotocin-induced diabetic rats in vivo. Extracts were administrated orally for two weeks at a dose of 60 mg/kg of body weight. In addition, the LDL level was also decreased [35]. Some members of the *Asteraceae* family also possess this ability. *Launaea taraxacifolia* leaf extract (20 μg/μL) was found to prevent lipid accumulation induced by oleic acid in HepG2 cells in vitro compared to controls [31] (Table 1).

Chicory supplementation can also reduce the hyperlipidemia. Keshk et al. [54] induced hyperlipidemia in rats with injection of Triton WR-1339, and next supplemented the rats with 10 g of chicory in 100 g of diet for 4 weeks. Obtained results showed the significant decrease in liver and heart acetyl-CoA carboxylase activity and improvement in lipid profile [54].

## 6. Other Cardioprotective Properties (Anti-Hypertension and Anti-Obesity) of Preparations from Plants of the *Asteraceae* Family and *Cucurbitaceae* Family

Hypertension, an increase in blood pressure caused by excessive contraction of the blood vessel, is a major risk factor for cardiovascular diseases. It has been associated with coronary diseases, cardiac arrhythmias, hypertrophy of left ventricular and cerebral stroke. In Europe, over 40% of the general population is believed to develop hypertension during their life [7,55].

Artichoke (*Cynara cardunculus*), a member of the *Asteraceae*, can lower blood lipid levels and inhibit cholesterol biosynthesis [7]. The extract can influence the gene expression of nitric oxide synthase in the endothelium and increase nitric oxide production in vascular endothelial cells. Roghani-Dehkordi et al. [56] demonstrated the artichoke’s ability to lower mild hypertension in otherwise healthy humans. In the study, 18% concentrate juice of artichoke in capsule was administrated orally to healthy male volunteers. After the 12 weeks the significant tension in volunteers was significantly lower than the baseline and also significantly lower compared with placebo group (Table 1) [7,56].

Oil from pumpkin seeds has also demonstrated antihypertensive activity, causing a significant decrease of nitric oxide level in serum in male Sprague–Dawley rats. In this case, hypertension was induced by the nitric oxide synthase inhibitor Nω-nitro-l-arginine methyl ester hydrochloride. Pumpkin seed oil was administrated orally at doses of 40 or 100 mg/kg of body weight (Table 1) [57]. The positive effect on pumpkin seeds oil on markers of cardiovascular diseases has been demonstrated also by Ramadan et al. [58]. The study evaluated the effect of pumpkin seeds oil on arterial blood pressure and cardiac functioning in rats fed a high fat diet. A total of 30 adult albino rats were divided into three groups, the first was control, second was treated with high fat diet, and the last was treated with pumpkin seeds oil. Pumpkin seeds oil significantly lower the body weight and blood pressure in rats. The level of LDL and triglycerides in serum were also decreased and inflammatory markers were suppressed (Table 1) [58].

Wahid et al. [59] analyzed the antihypertensive activity of *Cucumis sativus* ethanol extract based on blood pressure measurement and in vivo models of vasoconstriction in Sprague–Dawley rats. The extract consisted of 45% crude cucumber seeds dissolved in a mixture of ethanol and water. The cucumber seed extract was administrated orally in five dosages (50–300 mg/kg of body weight) each day for 28 days. The results indicate that the cucumber seeds demonstrated antihypertensive activity and were able to suppress inflammation with strong anti-myocardial infarction potential. The mechanism is based on the modulation of endothelium-derived relaxing factors (Table 1) [59].

One of the greatest risk factors in the development of CVD is obesity. Fortunately, many *Asteraceae* and *Cucurbitaceae* family members appear to have anti-obesity potential. For example, *Cynara scolymus*, of the *Asteraceae*, demonstrated diuretic and antihyperlipidemic potential, and may be used to control obesity (Table 1) [60].

In a study on the influence of chicory on obesity factors in vitro, Muthusamy et al. [61] found methanol extracts and detannified methanol extract of *C. intybus* leaves (1 pg/mL to 10 μg/mL) inhibit adiopogenesis in 3T3-L1 preadipocytes. Adipocyte differentiation was determined based on triglyceride content. Treatment was found to be associated with reduced triglyceride content in cells, suggesting anti-obesity activity [61].

Choi et al. [62] found water-soluble *Cucurbita moschata* extract to have anti-obesity potential in a mouse model in vivo. Briefly, 34 male mice were divided in two groups: the first (10 mice) received standard pelleted chow and the second (24 mice) a high fat diet. After six weeks, the second group was divided in two, and one subgroup was supplemented with *C. moschata* extract (500 mg/kg of body weight) once per day for eight weeks. After 8-weeks, although the overall amount of food intake was not affected, an increase in body weight and fat storage was suppressed. The *C. moschata* extract prevented the development of fatty liver and lowered the level of triglyceride and cholesterol in mouse blood, suggesting anti-obesity effect [62].

Ghahremanloo et al. [63] demonstrated that pumpkin can reduce dyslipidemia in obese rats, leading to the decrease of cardiovascular diseases risks. In a study, 30 male Wistar rats were divided into five groups, with healthy and dietary fatty controls and three experimental dietary fatty rats. The experimental groups received hydro-alcoholic extract of pumpkin at does 100, 200, and 400 mg/kg of body weight. After 6 weeks, researchers observed a huge decrease in low-density lipoproteins and triglycerides in the experimental groups. Additionally, the level of glutathione increased compared to the obese group (Table 1) [63].

This review demonstrated the promising studies on the effect of secondary metabolites from vegetables on cardiovascular diseases; however it also shows that studies are lacking on the effect of these compounds on metabolic markers in CVDs, such as body weight, blood pressure, glucose homeostasis, lipids, and biomarkers of inflammation. Although the health benefits of vegetables in diet are well-known, there is a lack of studies in the literature on their effect on cardiovascular diseases in a human model [8,11,12]. Most research on secondary metabolites from vegetables have not moved past in vitro studies. There are some in in vivo level as presented in Table 1, but only a few. There is only one example on studies on patients [59]. Therefore, further studies are needed to evaluate the effects of regular consumption of vegetables on CVDs and the risk factors for CVDs.

**Table 1:** Comparative in vitro and in vivo studies of cardioprotective activity of vegetables from *Cucurbitaceae* and *Asteraceae* families.

Vegetables	Cardioprotective Activity	Type of Solvent	Dose	Biological Material	Studies	References
	Antioxidant Activity					
*Cichorium intybus, Lactuca sativa, Helianthus tuberosus (Asteraceae)*	Scavenging free radical, inhibition of lipid peroxidation, and carbonylation of plasma protein	methanol	1–50 µg/mL	Human plasma	in vitro	[30]
*Cichorium intybus* (*Asteraceae*)	Scavenging free radicals	methanol	100 mg/mL	Human serum	in vitro	[27]
*Lactuca sativa, (Asteraceae*)	Scavenging free radicals	water	20 mg/mL	Human serum	in vitro	[20]
*Launaea taraxacifolia,* *(Asteraceae)*	Decrease in free radical production	ethanol-aqueous	1–20 μg/μL	PLB985 cells	in vitro	[31]
*Cucurbita pepo, Cucurbita pepo convar. Giromontina, Cucumis sativus, Cucurbita pepo var. patisoniana* (*Cucurbitaceae)*	Scavenging free radicals, inhibition of lipid peroxidation, and carbonylation of plasma protein	methanol	1–50 µg/mL	Human plasma	in vitro	[44]
*Momordica dioica* *(Cucurbitaceae)*	Scavenging free radicals	methanol/water (70%, 50%, 30%)	1–5000 µg/mL	-	in vitro	[47]
*Cucumis melo var. agrestis* *(Cucurbitaceae)*	Scavenging free radicals	methanol/water (70%, 50%, 30%)	1–5000 µg/mL	-	in vitro	[47]
*Citrullus colocynthus* *(Cucurbitaceae)*	Scavenging free radicals	methanol/water (70%, 50%, 30%)	1–5000 µg/mL	-	in vitro	[47]
*Momordica charantia* *(Cucurbitaceae)*	Inhibition of lipid peroxidation, scavenging free radicals	methanol, chloroform	100 mg/mL	Human plasma	in vitro	[48]
*Cichorium intybus* *(Asteraceae)*	Decrease in biomarkers of oxidative stress in blood	methanol	100%, 50%, 25%	Male albino Wistar rats	in vivo	[27]
*Cutrullus colocynthis* *(Cucurbitaceae)*	Decrease in lipid peroxidation biomarkers in liver tissue	no solvent/dry extract	300 mg/kg	Diabetic albino rats	in vivo	[49]
*Cucurbita ficifolia* *(Cucurbitaceae)*	Decrease in lipid peroxidation	water	300 mg/kg	Male Sprague–Dawley rats	in vivo	[50]
	Antiplatelet activity					
*Cichorium intybus, Lactuca sativa, Helianthus tuberosus (Asteraceae)*	Inhibition of adhesion of thrombin- and ADP-activated platelets to fibrinogen	methanol	1–50 µg/mL	Human blood platelets	in vitro	[33]
*Cucurbita pepo, Cucurbita pepo convar. Giromontina, Cucumis sativus, Cucurbita pepo var. patisoniana* (*Cucurbitaceae*)	Inhibition of adhesion of thrombin- and ADP-activated platelets to collagen and fibrinogen	methanol	1–50 µg/mL	Human blood platelets	in vitro	[51]
*Cichorium intybus* *(Asteraceae)*	Inhibition of platelet aggregation	water	300 mL of 20 g chicory coffee	Human	in vivo	[34]
*Lagenaria siceriaria* *(Cucurbitaceae)*	Inhibition of ADP-induced platelet aggregation	ethanol	250, 5000 and 1000 mg/kg	Swiss albino mouse	in vivo	[52]
	Anticoagulant activity					
*Cucurbita pepo var. patisoniana* *(Cucucrbitaceae)*	Inhibition of total thrombus formation	methanol	50 µg/mL	Whole blood	in vitro	[51]
	Anti-hyperlipidemic activity					
*Launaea taraxacifolia* *(Asteraceae)*	Inhibition of lipid accumulation	ethanol-aqueous	20 μg/μL	HepG2 cells	in vitro	[31]
*Cichorium intybus* *(Asteraceae)*	Improvement of lipid profile	no solvent	10 g/100 g of diet	male Wistar rats	in vivo	[54]
*Citrullus colocynthis* *(Cucurbitaceae)*	Decrease in serum triglyceride and cholesterol	methanol	60 mg/kg	Sprague–Dawley rats	in vivo	[59]
*Cucurbita moschata* *(Cucurbitaceae)*	Decrease in triglyceride and cholesterol in blood	ethanol-aqueous	500 mg/kg	Adult mice	in vivo	[62]
	Antihypertensive activity					
*Cucurbita pepo* *(Cucurbitaceae)*	Decrease of nitric oxide	no solvent	40 or 100 mg/kg of body weight	Sprague–Dawley rats (serum)	in vivo	[57]
*Cynara cardunculus (Asteraceae)*	Decrease in blood pressure	water	18% juice	Patients with mild-hypertension	in vivo	[56]
*Cucumis sativus* *(Cucurbitaceae)*	modulation of endothelium-derived relaxing factors	ethanol-aqueous	50–300 mg/kg	Sprague–Dawley rats	in vivo	[59]
	Anti-obesity and body weight control					
*Cichorium intybus* *(Asteraceae)*	Decrease in triglycerides content	methanol	1 pg/mL to 10 μg/mL	3T3-L1 preadipocytes cell	in vitro	[60]
*Cucurbita pepo* *(Cucurbitaceae)*	Decrease in LDL and triglycerides	methanol	100, 200, 400 mg/kg	male Wistar rats	in vivo	[63]
*Cucurbita pepo* *(Cucurbitaceae)*	Decrease in blood pressure, lower body weight, and LDL level	ethanol/methanol	100 mg/kg	male Wistar rats	in vivo	[58].

## 7. Toxicity of Vegetable Preparations from *Cucurbitaceae* and *Asteraceae* Families

Rolnik et al. [51] analyzed the cytotoxic activity of selected vegetables (pumpkin, zucchini, cucumber, white and yellow pattypan squash) from the *Cucurbitaceae* family on blood platelets in vitro. The cytotoxic effect was evaluated by level of extracellular lactate dehydrogenase. The obtained results indicate that the tested *Cucurbitaceae* vegetables had no cytotoxicity against blood platelets [51].

Moreover, Rolnik et al. [33] studied the cytotoxicity of extracts from four vegetables from the *Asteraceae* (chicory, Jerusalem artichoke, green and red lettuce) at a concentration of 50 μg/mL in vitro. None of tested preparations was found to cause blood platelet lysis, indicated by lactate dehydrogenase release from blood platelets [33].

## 8. Conclusions

For centuries, plants have been used as remedies. However, modern medicine often draws on ethnopharmacology to create innovative new treatment and there is consequently great interest in better understanding the mechanisms behind the biological activities and pro-health properties of plants. Such research is particularly valuable in cardiovascular diseases, when classic treatment is often associated with various side effects.

**Figure 1:**
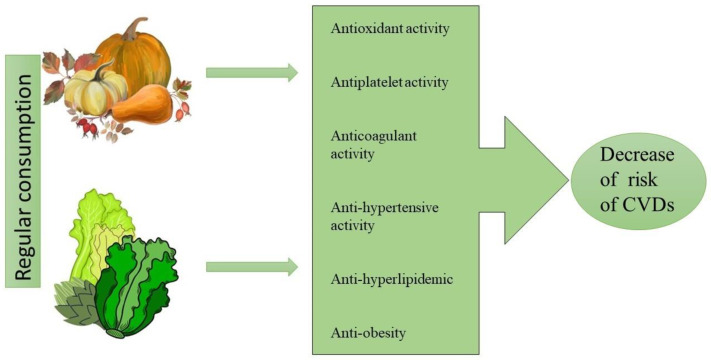
The effect of selected vegetables from *Cucurbitaceae* and *Asteraceae* family on development of cardiovascular diseases.

Research indicates that the consumption of vegetables from both *Asteraceae* and *Cucurbitaceae* may reduce risk of CVDs (Figure 1). Furthermore, their cardioprotective potential appears to often be correlated with their secondary metabolite content, making them attractive potential new sources of treatments for the prevention and treatment of cardiovascular diseases. However, as the available data from clinical experiments provide only a fragmentary insight into the cardioprotective potential of these vegetables and their safety, more extensive in vivo studies are required, particularly those involving human subjects, on vegetable preparations with well-known chemical characteristics.

## Data Availability

Not applicable.
